# Localised Autophagy Inhibition by Nanodiamonds Potentiates Arsenic Therapy With Favourable Safety Profile in Solid Tumours

**DOI:** 10.1111/cpr.70234

**Published:** 2026-05-19

**Authors:** Yiliu Wang, Zhifen Cui, Jichao Zhang, Shitai Zhu, Linjie Guo, Qisheng Wang, Ying Zhu, Shihua Luo, Huating Kong

**Affiliations:** ^1^ CAS Key Laboratory of Interfacial Physics and Technology, Shanghai Institute of Applied Physics, Chinese Academy of Sciences Shanghai China; ^2^ Institute of Materiobiology, College of Sciences Shanghai University Shanghai China; ^3^ University of Chinese Academy of Sciences Beijing China; ^4^ Shanghai Synchrotron Radiation Facility, Shanghai Advanced Research Institute, Chinese Academy of Sciences Shanghai China; ^5^ Department of Traumatology, Rui Jin Hospital, School of Medicine Shanghai Jiao Tong University Shanghai China

## Abstract

Arsenic trioxide (ATO) shows limited efficacy against solid tumours, largely because it induces protective autophagy that attenuates its pro‐apoptotic activity. Our previous studies established that nanodiamonds (NDs) function as nanoparticle autophagy inhibitors (NAPIs) when delivered systemically, markedly enhancing ATO efficacy in orthotopic liver tumour models by blocking NUPR1‐mediated autolysosomal clearance. However, systemic administration remains inefficient in modulating the local autophagic microenvironment within tumours. Here we developed an interventional strategy based on intratumoral injection to assess the feasibility and biosafety of locally blocking autophagic flux while substantially reducing the required ATO dosage, thereby maximising the synergistic anti‐tumour effects of NDs and ATO. In HepG2 hepatocellular carcinoma cells, NDs markedly blocked the late stage of autophagic flux, thereby significantly amplifying ATO‐induced apoptosis. In a subcutaneous xenograft liver cancer mouse model, intratumoral co‐administration of NDs with low‐dose ATO achieved ~91% tumour inhibition and effectively eliminated the systemic toxicity associated with high‐dose ATO monotherapy. Notably, the synergistic antitumor effect was independent of increased intratumoral ATO accumulation and was driven instead by targeted modulation of the autophagic pathway. Collectively, this study demonstrates a mechanism of localised, nanomaterial‐mediated autophagy regulation and offers an efficient, safe strategy for interventional therapy of advanced solid tumours.

## Graphical abstract

This study presents an NDs‐mediated localised combination strategy that integrates autophagy inhibition with low‐dose ATO sensitization, enabling effective tumour suppression while enhancing safety. NDs‐mediated autophagy blockade enhances ATO efficacy while minimising systemic toxicity, highlighting a promising approach for solid tumour treatment.

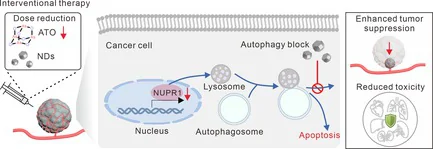

## 1 Introduction

Arsenic trioxide (ATO), a classical arsenical agent, has achieved remarkable clinical success in the treatment of acute promyelocytic leukaemia (APL) [1, 2, 3, 4, 5]. However, its efficacy against solid tumours remains limited, and durable tumour suppression is rarely achieved in malignancies with high tumour burden [6, 7, 8, 9, 10]. Accumulating evidence shows that, in solid tumour cells, ATO not only triggers apoptosis but also strongly induces protective autophagy, which alleviates cellular stress, clears damaged organelles, and thereby attenuates ATO‐induced cell death [11, 12, 13]. This “autophagy tolerance” mechanism is now recognised as a key barrier limiting the therapeutic efficacy of ATO in solid tumours. Accordingly, intervention against ATO‐induced protective autophagy has emerged as a promising strategy to improve its antitumor efficacy [14, 15].

In our previous work, we proposed and validated the concept of nanodiamonds (NDs) as nanoparticle autophagy inhibitors (NAPIs) [16]. When administered intravenously, NDs significantly potentiated ATO efficacy against orthotopic early‐stage liver tumours by blocking NUPR1‐mediated autolysosomal clearance. Subsequent work further demonstrated that this combination also effectively suppressed distant metastasis of liver cancer, highlighting its potential for anti‐metastatic therapy [17]. These studies collectively demonstrated, at both mechanistic and application levels, the feasibility of using nanomaterials to sensitise chemotherapy and suppress tumour progression through modulation of autophagic flux.

Nevertheless, systemic administration encounters substantial challenges in patients with advanced liver cancer, who typically present with large tumour volumes, hypervascularity, and compromised hepatic reserve. Aberrant tumour vasculature and elevated interstitial fluid pressure severely restrict nanomaterial penetration and homogeneous intratumoral distribution [18, 19, 20], while systemic dilution and rapid clearance further diminish local NDs activity within the tumour [21, 22, 23]. Moreover, patients with advanced disease often cannot tolerate the cumulative systemic toxicity of prolonged chemotherapy. In contrast, intratumoral injection offers superior local drug retention and minimises exposure of normal tissues, making it an attractive approach for intermediate and advanced liver cancer [24, 25]. However, the therapeutic window for intratumoral ATO remains poorly defined, and high local doses can still induce systemic toxicity [14, 26, 27].

In the current study, we developed an interventional strategy that combines NDs with low‐dose ATO delivered by intratumoral injection. This approach bypasses systemic delivery barriers, achieves efficient local drug exposure, and exerts synergistic antitumor effects while substantially lowering the required ATO dose. More importantly, the strategy amplifies low‐dose ATO efficacy through targeted blockade of protective autophagy rather than by increasing drug dosage. In the present study, we systematically evaluated the antitumor efficacy and biosafety of this localised NDs + ATO strategy in a subcutaneous xenograft liver cancer mouse model, providing a new theoretical and practical foundation for efficient interventional therapy of advanced solid tumours (Figure 1).

**FIGURE 1:**
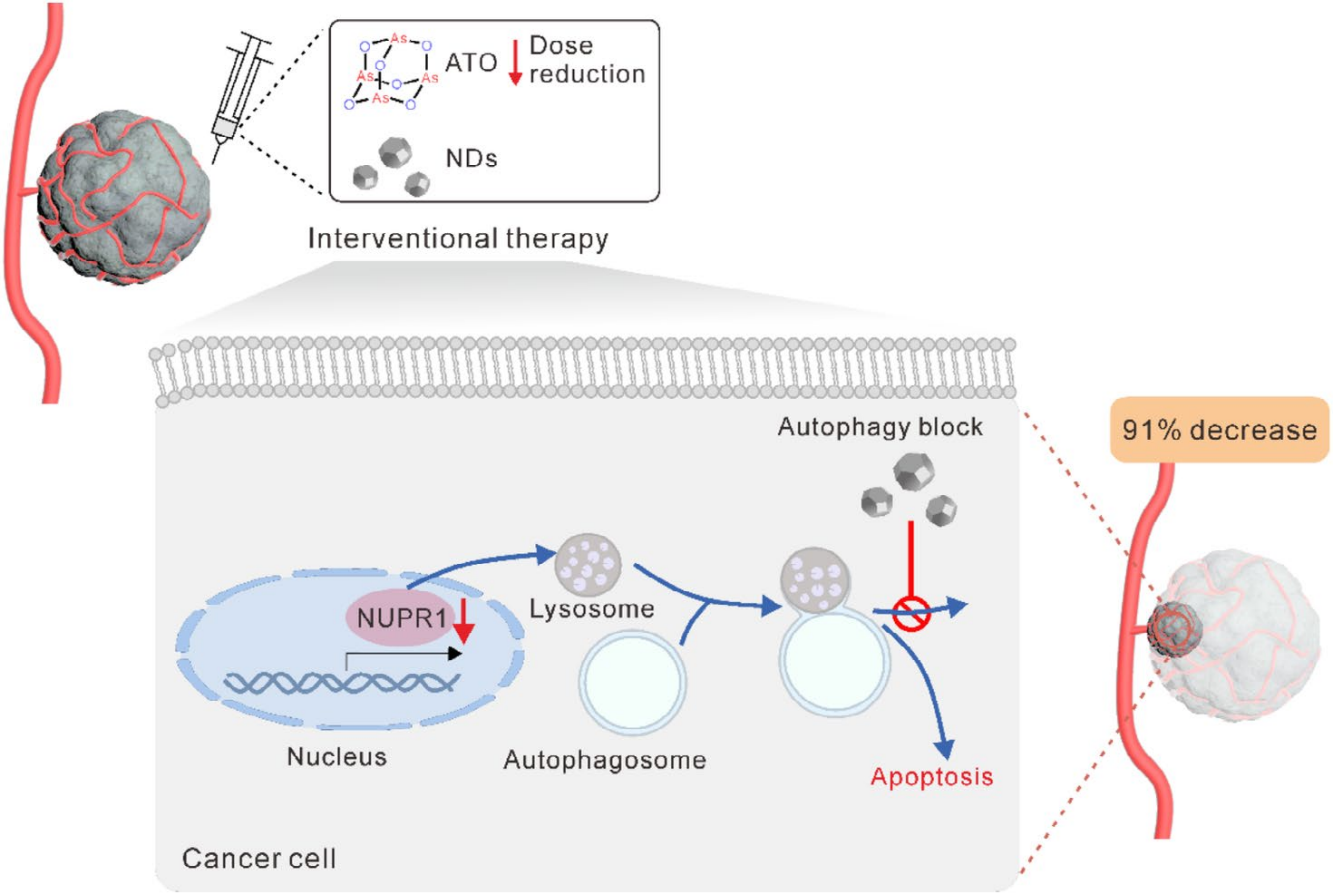
Schematic illustration of intratumoral NDs + ATO combination therapy. Intratumoral co‐administration of NDs and ATO enhances the antitumor effect by inhibiting autophagy, achieving a tumour inhibition rate of ~91%.

## 2 Materials and Methods

### 2.1 Materials

NDs with individual sizes of 2–10 nm produced by detonation technique were purchased from Gansu Gold Stone Nano. Material. Co. Ltd. (Gansu, China). An appropriate amount of NDs was weighed, resuspended in phosphate buffered saline (PBS), and sonicated in ultrasonic disruptor for 15 min (amplitude 100 A, 5 s pulse, 5 s pause) to obtain a well‐dispersed suspension. ATO was purchased from Sigma‐Aldrich, USA. A stock solution of ATO was prepared with 0.1 M NaOH and was used to make serial dilutions.

### 2.2 Cell Culture and Treatment

HepG2 liver carcinoma cells (ATCC) were grown in RPMI1640 (Gibco) cell culture medium supplemented with 10% foetal bovine serum (FBS). The resultant cell suspension (7 × 10^4^ cells mL^−1^ or 4 × 10^5^ cells mL^−1^) was dispensed into 24‐well plates or 6‐well plates and incubated overnight. After washing twice with PBS, cells were exposed to NDs (50 μg mL^−1^), ATO (4 μM), and NDs + ATO mixture for 48 h as required. Then, the cells were collected for the following experiments: (1) CCK8 assay, (2) flow cytometry, (3) western blotting analysis, and (4) evaluation of dysfunction in autolysosomal processing.

### 2.3 CCK8 Assay

Cell viability was determined by CCK8 assay (Beyotime) according to the manufacturer's instructions. In brief, drug treatment was carried out for 48 h, CCK‐8 (reagent: culture medium = 1:10) was added into each well and cultured in the dark at 37°C for 1 h. Absorbance was read at 450 nm. Cells treated without drugs were used as control.

### 2.4 Flow Cytometry

Apoptotic cells were stained using the Annexin V‐FITC Apoptosis Detection Kit (Beyotime) and analysed by flow cytometry. Drug treatment was carried out for 48 h, the medium was collected and washed with pre‐cooled PBS before digestion with trypsin without EDTA. After centrifugation, cells were counted and resuspended in Annexin V‐FITC binding solution and stained with Annexin V‐FITC and PI staining solution. Cells were stained at room temperature in the absence of light for 20 min and immediately analysed via flow cytometry (BD Biosciences, FACSymphonyA1).

### 2.5 Evaluation of Dysfunction in Autolysosomal Processing

After 24 h transfection of the cells with mRFP‐GFP‐LC3 plasmid (Addgene), cells were further treated with NDs (50 μg mL^−1^), ATO (4 μM) or NDs + ATO mixture for 48 h. The cells were washed with PBS and imaged using a confocal laser scanning microscope (Leica Microsystems, TCS sp8). Select 10 cells from each group for statistical analysis.

### 2.6 Tumour Models and Treatment

Nude mice (male, 20 g) were purchased from Shanghai SLAC Laboratory Animal Co. Ltd., China and raised at 22°C under pathogen‐free (SPF) conditions. All animal experiments were conducted in accordance with the Institute's Guidelines for the Welfare and Use of Laboratory Animals and were approved by the Ethics Committee of Shanghai Beautiful Life Medical Technology Co Ltd. (Approval No.: SYXK‐2017‐0016, Approval Date December 25, 2017).

For the subcutaneous xenograft tumour models, HepG2 cells (2 × 10^6^) in 200 μL saline were injected subcutaneously into the right forelimbs of nude mice [28]. After 7 days, mice were intratumorally injected with 100 μg NDs, 20 μg ATO, a mixture of 100 μg NDs and 20 μg ATO, 80 μg ATO, 200 μg ATO, or 100 μL of (normal saline, NS) (six per group) once a day for 15 consecutive days. After the final injection, all of the mice were sacrificed at day 22 for the following experiments: (1) antitumor response evaluation, (2) biocompatibility assessment, (3) metallomics imaging, and (4) autophagy assessment.

The serum samples were collected for biochemical assays. Levels of ALT and AST activities, Crea, BUN and UA concentrations were determined according to the detection kit (Nanjing Jiancheng Bioengineering Institute, China). After weighing the body and tissues, the coefficients of heart, liver, spleen, lung and kidney to body weight were calculated as the ratio of tissues (wet weight, mg) to body weight (g).

### 2.7 Western Blotting Analysis

At 48 h following drug treatment, cells were lysed in SDS‐loading buffer and subjected to SDS‐PAGE followed by transfer onto PVDF membranes. The blots were blocked for 30 min with blocking solution and then incubated overnight at 4°C with the primary antibodies as required: anti‐LC3B (1:1000 diluted, Novus), anti‐P62 (1:1000 diluted, Abcam), anti‐NUPR1 (1:1000 diluted, Abcam) or anti‐GAPDH (1:1000 diluted, Abcam). After three washes in PBST (PBS containing 0.1% Tween 20), the membranes were incubated with goat anti‐rabbit/mouse horseradish peroxidase‐conjugated antibody (1:10000 diluted, KPL) for 1 h. The membrane was incubated with chemiluminescence (ECL) substrate, and then exposed to film, which was developed and scanned. The densities of all bands were quantified with a computer densitometer (AlphaImager 2200 System Alpha Innotech Corporation, GBBOX‐Chemi‐XL1.4). The expression of GAPDH was used as a control for protein loading.

The tumour tissues were chopped and homogenised in lysis buffer. The lysates were centrifuged to remove the debris, and the levels of LC3, p62, and NUPR1 in lysates were measured as above.

### 2.8 Immunostaining

All paraffin‐embedded tissue sections were repaired in the improved citrate antigen retrieval solution (Beyotime) at 100°C for 30 min. After two washes with PBS, sections were treated with 6% BSA, 0.25% Triton X‐100 in PBS for 45 min at room temperature. Then, sections were stained with rabbit anti‐LC3 antibody (1:200 diluted, Novus) or rabbit anti‐Atg13 antibody (1:200 diluted, Cell Signalling) overnight at 4°C, followed by Alexa Fluor 488 goat anti‐rabbit secondary antibody (1:300 diluted, Cell Signalling) at 37°C for 50 min. The stained tissue sections were visualised under a laser confocal microscope (Leica Microsystems, TCS sp8).

### 2.9 Synchrotron‐Based X‐Ray Fluorescence Microscopy

Synchrotron‐based X‐ray fluorescence (XRF) microscopy was used to investigate the metallomics of tumour after intratumoral injection. Paraffin‐embedded subcutaneous tumour tissue sections were placed on Mylar X‐ray films and the XRF microscopy was performed at the beamline BL15U1 of SSRF. Incident X‐ray energy was 12 keV, spot size was 100 × 100 μm, and data collection time for each pixel was 2 s. From the analysis of the X‐ray fluorescence spectrum for each pixel, a spatial image can be obtained for each element separately. Fitting of the fluorescence data was performed in batch processing using PyMca 4.0.9 software.

### 2.10 Transmission Electron Microscopy

Tumour tissues were immediately fixed in 2.5% glutaraldehyde in 0.1 M phosphate buffer (PB) and stored at 4°C until embedding. Then the tissues were embedded in a resin matrix to prepare ultrathin sections. Ultrathin sections of the tumour tissues were examined by transmission electron microscope (TEM, JEOL‐1230).

### 2.11 Histopathological Analysis and TUNEL Analysis

Heart, liver, spleen, lung, kidney, intestine, and tumour tissues were fixed in 4% paraformaldehyde in PBS, paraffin embedded, sectioned, and stained with haematoxylin and eosin (H&E) for histological analysis. The pathologist performing the visual analysis was blind to the grouping.

Terminal deoxynucleotidyl transferased UTP nick end labelling (TUNEL) assay was performed using in situ death‐detection POD kit (Roche Diagnostics). In short, sections were dewaxed in xylene and rehydrated with decreasing concentrations of ethanol, followed by labelling DNA strand breaks according to the manufacturer's instructions. Results were analysed by confocal microscopy (Leica Microsystems, TCS sp8) and quantified with ImageJ (NIH).

### 2.12 Inductively Coupled Plasma‐Mass Spectrometry (ICP‐MS)

After treatment, subcutaneous tumour tissue of mice was weighed and digested by HNO_3_ and H_2_O_2_ mixture (v/v ratio is 7:1) at 180°C until the mixed solutions became colourless and clear. As concentrations in tumour tissues were analysed by ICP‐MS.

### 2.13 Statistical Analysis

All results are expressed as the mean ± SD from triplicate experiments performed in a parallel manner unless otherwise indicated. Statistical significance of the data was determined by *t*‐tests or one‐way analysis of variance (ANOVA) using SPSS. *p < 0.05; **p < 0.01; ***p < 0.001.

## 3 Results

### 3.1 NDs Inhibit Autophagic Flux and Enhance ATO‐Based Apoptosis at the Cellular Level

To elucidate the synergistic antitumor effects of NDs and ATO at the cellular level, we first assessed cell viability and apoptosis in HepG2 cells under various treatment conditions (Figure 2a). CCK‐8 assays revealed that ATO alone significantly reduced cell viability relative to the control, whereas NDs alone produced negligible cytotoxicity. Notably, the NDs + ATO combination further decreased viability compared with ATO monotherapy, indicating synergistic cytotoxicity (Figure 2b). Flow‐cytometric analysis using Annexin V‐FITC/PI double staining showed apoptotic rates of 1.7%, 3.74%, and 10.45% in control, NDs, and ATO groups, respectively. In contrast, the NDs + ATO group exhibited an apoptotic rate of 24.29%, representing a 6.5‐fold increase over NDs alone and a 2.3‐fold increase over ATO alone, confirming that NDs markedly amplified ATO‐induced apoptosis (Figure 2c). We next examined the impact of these treatments on intracellular autophagic flux using western blotting and the tandem mRFP‐GFP‐LC3 reporter system. Western blotting was used to detect the phosphatidylethanolamine‐conjugated autophagy marker LC3, the autophagy substrate p62 [29, 30, 31] and the transcriptional regulator NUPR1 [32, 33]. ATO treatment alone increased LC3‐II and NUPR1 levels while reducing p62 expression, indicating enhanced autophagic flux. By contrast, the NDs + ATO group showed LC3‐II accumulation, increased p62, and downregulation of NUPR1, suggesting blockade of late‐stage autophagic flux (Figure 2d). mRFP‐GFP‐LC3 imaging showed that ATO alone led to an increase of yellow puncta (mRFP‐GFP‐LC3‐positive autophagosomes), characteristic of the increase in autophagosome‐lysosome flow. Whereas NDs + ATO treatment led to accumulation of red puncta (mRFP‐positive, GFP‐fluorescence‐negative autolysosomes), demonstrating that NDs prevent autolysosomal clearance (Figure 2e). Together, these results establish that, under ATO‐induced autophagic stress, NDs block late‐stage autophagic flux via NUPR1 downregulation and thereby convert the protective autophagic response into enhanced apoptosis.

**FIGURE 2:**
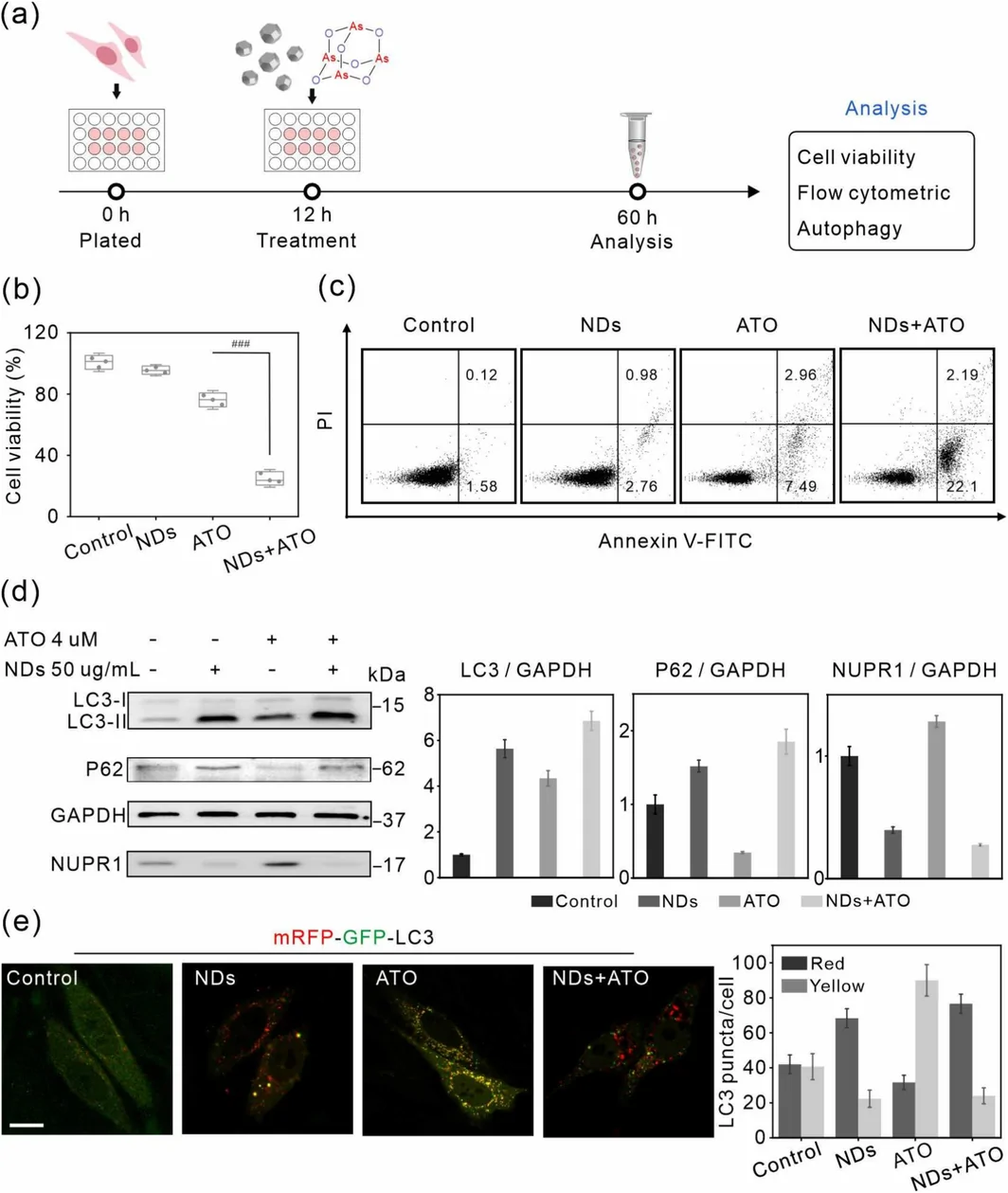
Assessment of apoptosis and autophagy at the cellular level. (a) Schematic illustration of cell seeding, drug treatment, and experimental design. (b) Comparison of viability of HepG2 cells after incubation with NDs, ATO, or NDs + ATO mixture for 48 h (*n* = 3). Data represented as mean ± SD. ^###^ *p* < 0.001 by t‐test, indicated a significant difference between the ATO group and the NDs + ATO group. (c) Apoptosis analysis of HepG2 cells by Annexin V‐FITC/PI double staining after 48 h treatment. Annexin V‐positive and PI‐positive cells were considered apoptotic cells in the total population. (d) Left: Immunoblots of autophagy related proteins LC3‐II, p62, and NUPR1. Right: Semi‐quantified analysis of cells treated with PBS, NDs, ATO, or NDs + ATO. Data represented as mean ± SD. (e) Representative fluorescence images of mRFP‐GFP‐LC3 cells after 48 h treatment with PBS, NDs, ATO, or NDs + ATO (autophagosomes, yellow puncta; autolysosomes, red puncta), together with quantification of LC3 puncta per cell. Data are presented as mean ± SD. Scale bar, 10 μm.

### 3.2 NDs Combined With Low‐Dose ATO via Intratumoral Injection Exhibit Significant Synergistic Anti‐Tumour Effects

Our previous work has demonstrated that nanodiamonds exhibit excellent biocompatibility when applied as autophagy inhibitors in tumour therapy [34, 35, 36, 37]. Intratumoral administration of ATO can substantially restrict drug distribution to non‐target organs, thereby permitting higher local doses while enhancing antitumour efficacy. Prior studies have reported varying degrees of tumour suppression in solid tumours with intratumoral ATO, yet high‐dose ATO is limited by pronounced dose‐dependent toxicity [38, 39, 40, 41]. To identify a safe and effective ATO dose for the NDs + ATO intratumoral injection strategy, we evaluated different ATO doses in a subcutaneous xenograft mouse model. Tumour‐bearing mice received daily intratumoral injection of normal saline (NS), NDs, ATO (1 mg/kg), NDs + ATO mixture (ATO at 1 mg/kg), ATO (4 mg/kg), or ATO (10 mg/kg) for 15 consecutive days (Figure 3a). ATO monotherapy exerted significant antitumour activity only at doses of 4 mg/kg or higher (Figure 3b). However, haemoglobin levels in the high‐dose ATO groups (4 and 10 mg/kg) decreased in a dose‐dependent manner, indicating a risk of hematologic toxicity (Figure 3c). In contrast, low‐dose ATO (1 mg/kg) combined with NDs achieved tumour inhibition comparable to high‐dose (4 and 10 mg/kg) ATO monotherapy, with haemoglobin levels indistinguishable from the normal control group (Figure 3b,c). These dose‐screening results clearly demonstrate that NDs not only substantially reduce the effective therapeutic dose of ATO but also effectively circumvent the hematologic toxicity associated with dose accumulation. Further biosafety evaluation showed no significant differences in the coefficients of major organs among any treatment groups relative to the NS group (Figure S1), indicating that organ weights remained within the normal range. H&E staining of the heart, liver, spleen, lung, kidney, and intestine revealed no overt histopathological abnormalities, indicating that NDs combined with low‐dose ATO caused no detectable organ damage (Figure S2). In addition, serum markers of liver function (ALT, AST) and renal function (Crea, BUN, UA) remained within normal ranges across all groups (Figure 3d,e). Taken together with the haemoglobin results, these findings demonstrate that NDs combined with low‐dose ATO achieves robust tumour inhibition while avoiding the dose‐dependent adverse effects of high‐dose ATO, thereby offering a markedly improved therapeutic window.

**FIGURE 3:**
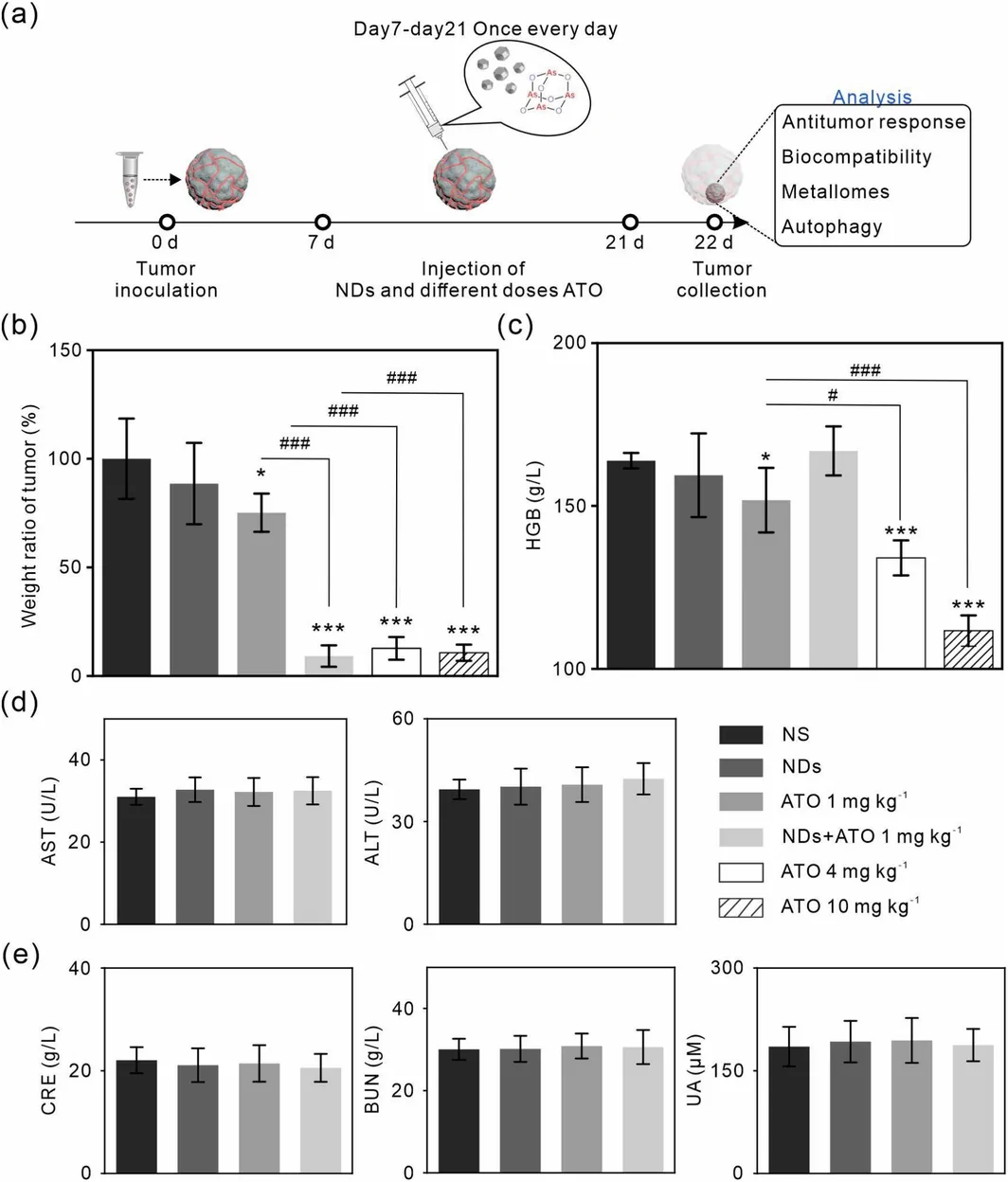
Biosafety assessment of intratumoral combination therapy of NDs and low‐dose ATO. (a) Schematic illustration of tumour model establishment, treatment and experimental design. (b) Tumour weight ratios in different treatment groups after 15 days of administration. *p < 0.05 and ***p < 0.001 versus NS group; ^###^ *p* < 0.001 versus ATO group by *t*‐test. (c) Haemoglobin levels in the blood of mice after different treatments. *p < 0.05 and ***p < 0.001 versus NS group; ^#^ *p* < 0.05 and ^###^ *p* < 0.001 versus ATO group by *t*‐test. (d) Serum biochemical parameters associated with liver injury after different treatments. (e) Serum biochemical parameters associated with kidney injury after different treatments. Data are presented as mean ± SD.

We next systematically evaluated the in vivo antitumor efficacy of intratumoral NDs combined with low‐dose ATO in the same subcutaneous xenograft model. Tumour growth curves showed that NDs alone exerted limited inhibitory effects, whereas ATO monotherapy only modestly delayed tumour growth. In contrast, the NDs + low‐dose ATO combination markedly suppressed tumour growth (Figure 4a). After 15 days of treatment, tumour weight in the ATO monotherapy group was reduced to 75% of that in the NS control, while NDs alone showed no significant effect. Remarkably, co‐administration of NDs and low‐dose ATO further reduced tumour weight to 9% of the NS control value, corresponding to a tumour inhibition rate of 91% and demonstrating pronounced synergistic antitumor activity in vivo (Figure 4b, Figure S3). TUNEL staining revealed a substantial increase in TUNEL‐positive cells in the NDs + low‐dose ATO group compared with ATO monotherapy. Quantitative analysis showed that the proportion of apoptotic cells rose from 18% to 32% in the combination group (Figure 4c). Consistent with these results, H&E staining of tumour sections from the NDs + low‐dose ATO group exhibited markedly more apoptotic cells (red arrows) than those from the ATO group (Figure 4d). Collectively, TUNEL and H&E analyses confirmed the enhanced pro‐apoptotic effect of the combined treatment. These data demonstrate that intratumoral co‐administration of NDs and low‐dose ATO potently enhances the antitumor activity of ATO, achieving ~91% tumour inhibition in the subcutaneous xenograft model. This pronounced synergy indicates that local blockade of protective autophagy effectively redirects ATO‐induced cellular stress into an irreversible apoptotic programme, thereby substantially improving therapeutic efficacy against solid tumours.

**FIGURE 4:**
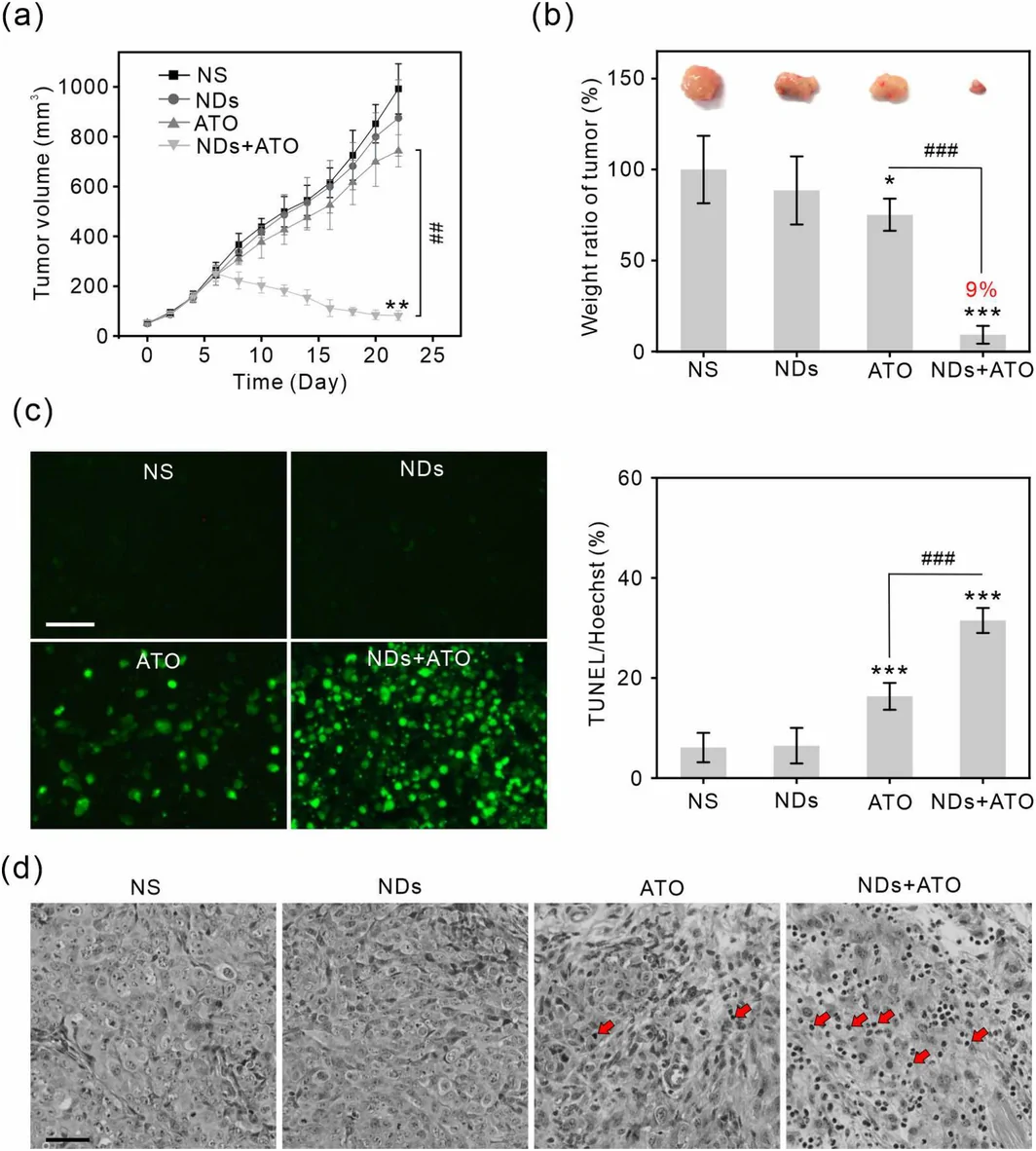
NDs NAPI improves low‐dose ATO based therapy in tumour bearing mouse model. (a) Tumour growth curves in different treatment groups. Data are presented as mean ± SD. **p < 0.01 versus NS group; ^##^ *p* < 0.01 versus ATO group by *t*‐test. (b) Tumour weight ratios and corresponding images of excised tumours after 15 days of administration. Ratios were normalised to the NS‐treated group. Data are presented as mean ± SD. *p < 0.05 and ***p < 0.001 versus NS group; ^###^ *p* < 0.001 versus ATO group by *t*‐test. (c) Representative TUNEL staining images and quantitative analysis by ImageJ. Scale bar, 100 μm. Data are presented as mean ± SD. ***p < 0.001 versus NS group; ^###^ *p* < 0.001 versus ATO group by *t*‐test. (d) Representative H&E‐stained tumour sections. Scale bar, 50 μm. Apoptotic cells are indicated by red arrows. The ATO dose was 1 mg/kg in all ATO‐containing groups.

### 3.3 Local Distribution of NDs + ATO After Intratumoral Injection

To determine whether NDs enhanced therapeutic efficacy by increasing ATO accumulation within tumour tissue, we mapped the spatial distribution of arsenic (As) and selected endogenous trace elements using synchrotron‐based X‐ray fluorescence (XRF) microscopy (Figure 5a). XRF imaging revealed no significant difference in either the distribution range or signal intensity of As between the NDs + ATO group and the ATO‐monotherapy group (Figure 5b,c). Quantitative inductively coupled plasma‐mass spectrometry (ICP‐MS) analysis of tumour tissues collected on day 15 confirmed that intratumoral As concentrations were statistically indistinguishable (1431 ± 185 ng/g in the NDs + ATO group versus 1445 ± 200 ng/g in the ATO group; *p* > 0.05; Figure 5d). In addition, XRF analysis revealed no significant alterations in the levels or spatial distributions of endogenous trace elements (Fe, Cu, Zn) across treatment groups, indicating that NDs did not disturb trace‐element homeostasis within the tumour (Figure 5b,c). These findings collectively indicate that the enhanced efficacy of NDs in ATO‐based interventional therapy is independent of increased intratumoral ATO accumulation.

**FIGURE 5:**
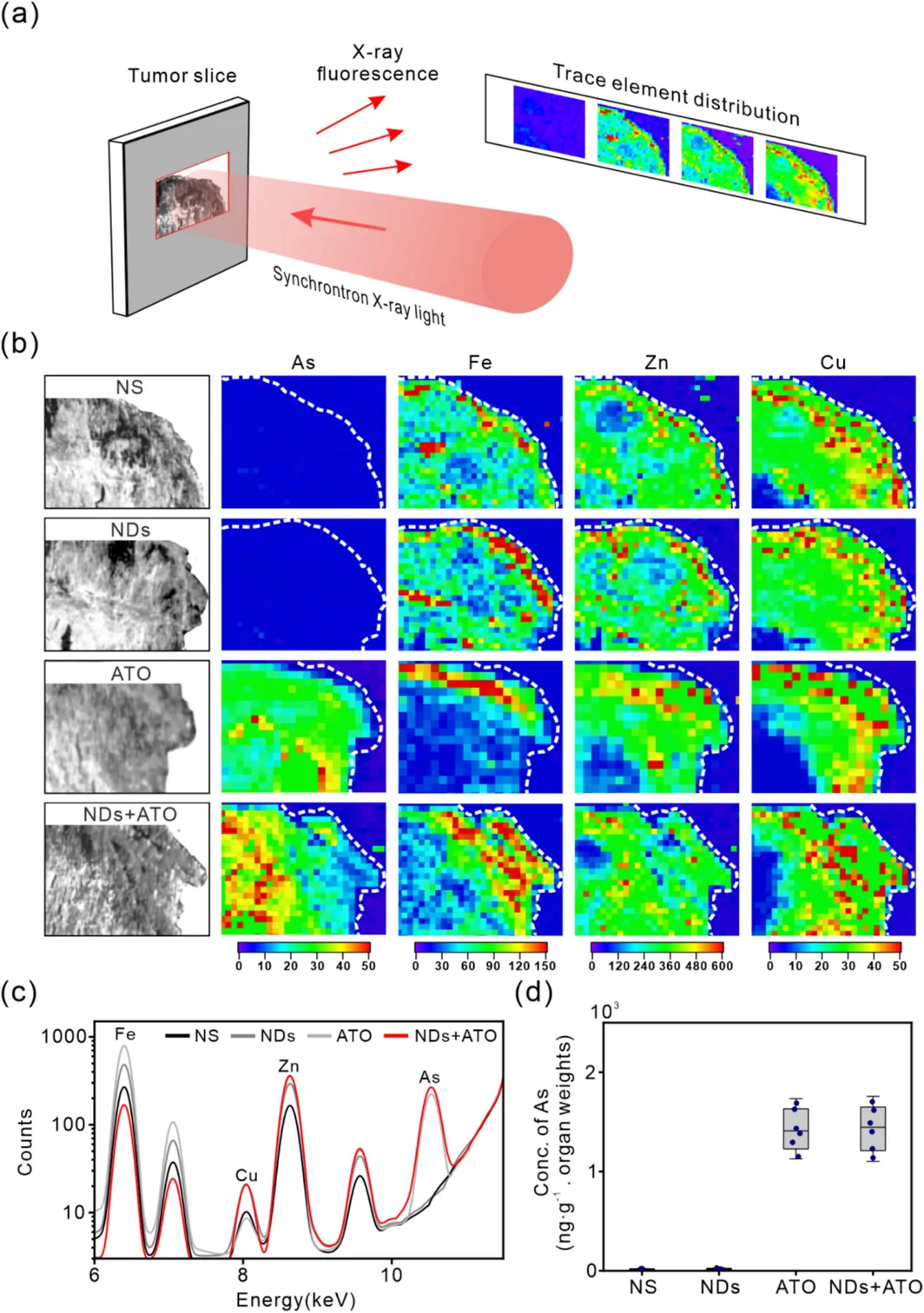
The elements distribution in tumour. (a) Schematic illustration of trace‐element biodistribution analysis by XRF. (b) Distribution of As, Fe, Cu, and Zn in tumour tissues collected on day 22. Each XRF image (right) is paired with the corresponding histological image (left). (c) Elemental spot spectra of As, Fe, Cu, and Zn in tumour tissues collected on day 22. (d) As concentration in tumour tissues on day 22 determined by ICP‐MS. Data are presented as mean ± SD.

### 3.4 Mechanistic Validation of Autophagic Flux Blockade by NDs + ATO Combination Therapy

Previous studies established that NDs sensitise ATO therapy against orthotopic early‐stage liver tumours under intravenous administration by inhibiting autophagy [16, 17, 42]. In the present study, we further showed that the antitumor effect of intratumoral NDs + ATO was independent of increased ATO accumulation within tumour tissue. We therefore hypothesised that the observed synergy mainly resulted from local inhibition of tumour autophagy by NDs. To test this hypothesis, we systematically investigated the molecular mechanism underlying NDs‐mediated potentiation of ATO activity in vivo. Western blot analysis revealed pronounced LC3‐II accumulation and p62 stabilisation in tumour tissues of the NDs + ATO group relative to ATO monotherapy (Figure 6a), indicating blockade of late‐stage autophagic flux. Immunofluorescence staining for the autophagic markers LC3 and ATG13 [43, 44, 45] further confirmed this blockade, with markedly elevated fluorescence signals observed in the NDs + ATO group (Figure 6b). Western blotting additionally showed significant downregulation of NUPR1 protein levels in the NDs + ATO group, which coincided with p62 accumulation (Figure 6c, left). Transmission electron microscopy (TEM) provided ultrastructural validation: ATO monotherapy induced abundant autophagosomes (red arrows), whereas NDs + ATO treatment markedly reduced autophagosome numbers while promoting accumulation of NDs‐containing autolysosomes (Figure 6c, right), demonstrating interference with autolysosomal maturation and clearance. Taken together, these results establish that NDs act as NAPIs by suppressing NUPR1‐mediated autolysosomal clearance, thereby converting ATO‐induced protective autophagy into a potent pro‐apoptotic signal and yielding markedly enhanced therapeutic efficacy against liver tumours.

**FIGURE 6:**
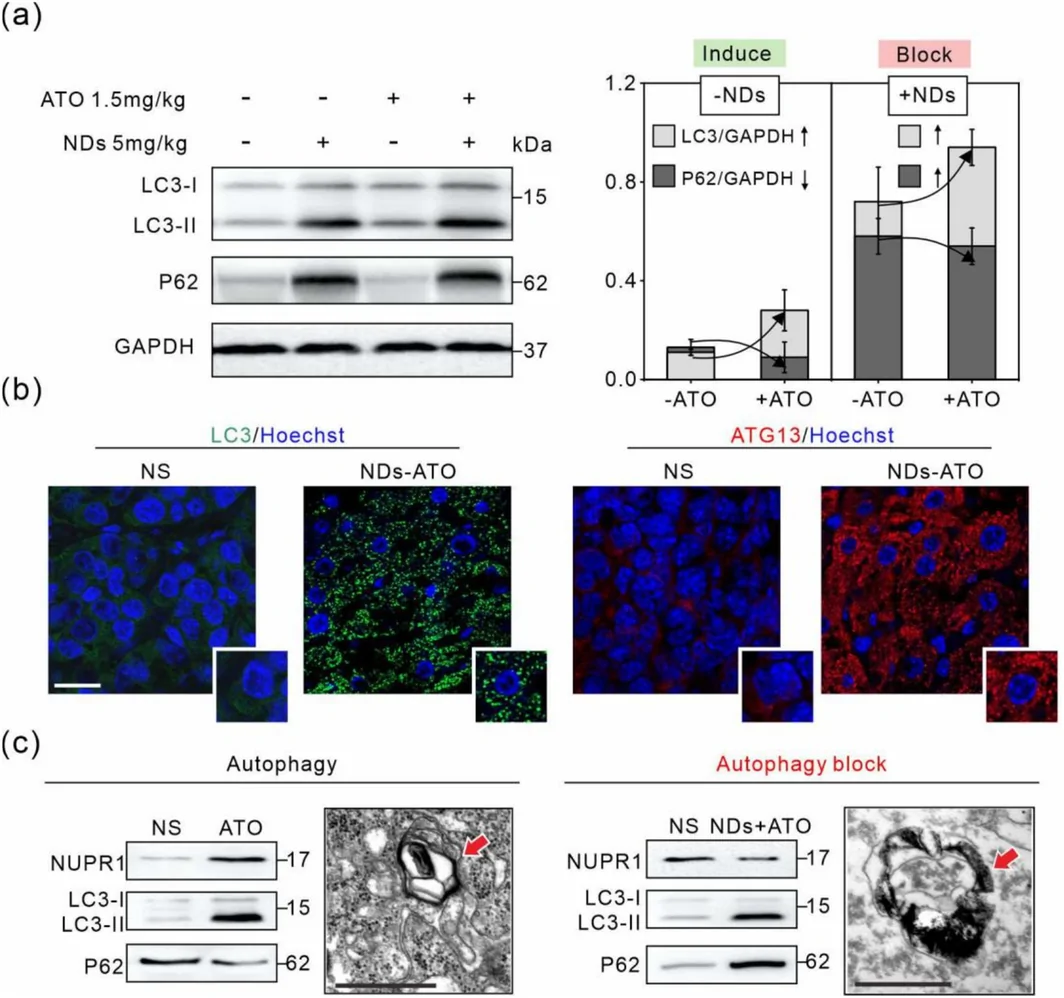
Molecular mechanism for NDs API‐enhanced ATO therapy of liver tumours. (a) Left: Immunoblots of LC3‐II and p62. Right: Semi‐quantitative analysis of tumour tissues after treatment (*n* = 3). GAPDH was used as the loading control. Data are presented as mean ± SD. (b) Immunostaining of LC3 and ATG13 in tumour tissues from mice treated with NDs + ATO. Scale bar, 10 μm. (c) Left: Immunoblots of LC3‐II and the autolysosome‐related protein NUPR1. GAPDH was used as the loading control. Right: TEM images of tumour tissues from mice treated with ATO or NDs + ATO. Scale bar, 1 μm. Representative autophagosomes induced by ATO and NDs‐containing autolysosomes induced by NDs + ATO are indicated by red arrows.

## 4 Discussion

To overcome the limited ability of systemic ATO administration to regulate the local autophagic microenvironment within solid tumours, we developed an intratumoral co‐administration strategy combining NDs with low‐dose ATO. Our results demonstrate that NDs do not increase intratumoral ATO accumulation; instead, they suppress autophagic flux through NUPR1 downregulation and redirect ATO‐induced protective autophagy towards apoptosis. Consequently, the combination markedly enhances antitumor activity while substantially reducing the required ATO dose, thereby eliminating the hematologic toxicity and major‐organ damage associated with high‐dose ATO. These findings highlight the unique advantage of localised, nanomaterial‐mediated autophagy regulation in potentiating low‐dose chemotherapy.

Compared with our previous studies based on systemic administration [16, 17], the local delivery of chemotherapeutic agents combined with modulators of autophagy can improve the efficacy of localised chemotherapy while also promoting systemic antitumor immune responses. Previous studies have demonstrated that such strategies not only effectively suppress the growth of primary tumours, including malignant melanoma and breast cancer, but also inhibit the progression of untreated contralateral tumours, indicating a potential abscopal effect [46]. Similarly, the present work optimises the delivery route and shows that intratumoral injection bypasses vascular barriers and systemic clearance, enabling local enrichment of NDs, efficient autophagy blockade, and robust apoptosis induction within the tumour. The NDs + low‐dose ATO interventional strategy proposed here achieves a more favourable balance between efficacy and safety, broadens the therapeutic window of ATO, and offers clear translational potential. More broadly, this study introduces a new paradigm for combining interventional therapy with NAPIs: localised regulation of autophagic pathways by NAPIs may minimise systemic exposure while maximising local efficacy, stability, and safety, thereby advancing efficient treatment of intermediate and advanced solid tumours.

Given the pleiotropic nature of autophagy‐related markers, our investigation of the autophagic pathway remains somewhat limited. Future studies should employ long‐term live‐cell fluorescence tracing and other approaches to further validate autophagic flux dynamics. Although the subcutaneous xenograft model is one of the most widely used models for hepatocellular carcinoma, it does not fully recapitulate the complex microenvironment of orthotopic liver tumours. In addition, this ectopic model has inherent limitations in distinguishing the spatial organisation and morphological interactions between tumour cells and the surrounding stroma. The current model should be extended to orthotopic or immunocompetent models, with further optimisation of dosing frequency and NDs particle size, as well as exploration of combination regimens with other therapeutic modalities, to facilitate clinical translation. Looking ahead, this work not only expands the application scope of NDs as functional nano‐regulatory materials but also suggests that the use of NAPIs for localised and efficient tumour intervention may represent a broadly applicable strategy to improve the efficacy of arsenicals and other stress‐inducing anticancer agents in solid tumours. Overall, our study provides a new route towards efficient interventional therapy of advanced solid tumours with both practical feasibility and high safety.

## Author Contributions

Yiliu Wang, Zhifen Cui, and Jichao Zhang performed the experiments. Shitai Zhu assisted in the biosafety assessment. Linjie Guo assisted in flow cytometry analysis. Qisheng Wang performed critical revisions. Yiliu Wang, Zhifen Cui, Ying Zhu, Shihua Luo, and Huating Kong designed the experiments, analysed the data, and wrote versions of the manuscript. All authors discussed and commented on the manuscript.

## Funding

This work was supported by National Key R&D Program of China, 2022YFA1603600. National Natural Science Foundation of China, 22525605, 32301185, 22274097. Natural Science Foundation of Shanghai, 23ZR1471200.

## Conflicts of Interest

The authors declare no conflicts of interest.

## Supporting information


**Figure S1:** Coefficients of vital organs in subcutaneous xenograft mice after different treatments.
**Figure S2:** The HE staining of vital organs in subcutaneous xenograft mice after different treatments. Scale bar: 100 μm.
**Figure S3:** Images of excised tumours in different treatment groups.

## Data Availability

The data that support the findings of this study are available from the corresponding author upon reasonable request.
