# Supplementary material for: Localised Autophagy Inhibition by Nanodiamonds Potentiates Arsenic Therapy With Favourable Safety Profile in Solid Tumours
Source: Cell Prolif. 2026 May 19:e70234. Online ahead of print. doi: 10.1111/cpr.70234 (PMC13325723; doi:10.1111/cpr.70234)
Supplement: Supplementary file 1 — Figure S1: Coefficients of vital organs in subcutaneous xenograft mice after different treatments. Figure S2: The HE staining of vital organs in subcutaneous xenograft mice after different treatments. Scale bar: 100 μm. Figure S3: Images of excised tumours in different treatment groups. [file CPR-9999-e70234-s001.docx]

**Supporting Information**

Localized autophagy inhibition by nanodiamonds potentiates arsenic therapy with favorable safety profile in solid tumors

Yiliu Wang^1,2,3#^, Zhifen Cui^1#^, Jichao Zhang^4#^, Shitai Zhu^1,2,3^, Linjie Guo^2^, Qisheng Wang^4^, Ying Zhu^2,3^, Shihua Luo^5*^, Huating Kong^4*^

^1.^ CAS Key Laboratory of Interfacial Physics and Technology, Shanghai Institute of Applied Physics, Chinese Academy of Sciences, Shanghai 201800, China

^2.^ Institute of Materiobiology, College of Sciences, Shanghai University, Shanghai 200444, China

^3.^ University of Chinese Academy of Sciences, Beijing 100049, China

^4.^ Shanghai Synchrotron Radiation Facility, Shanghai Advanced Research Institute, Chinese Academy of Sciences, Shanghai 201204, China

^5.^ Department of Traumatology, Rui Jin Hospital, School of Medicine, Shanghai Jiao Tong University, Shanghai 200025, China

**Corresponding Authors**

* E-mail: konght@sari.ac.cn

* E-mail: jqab@163.com


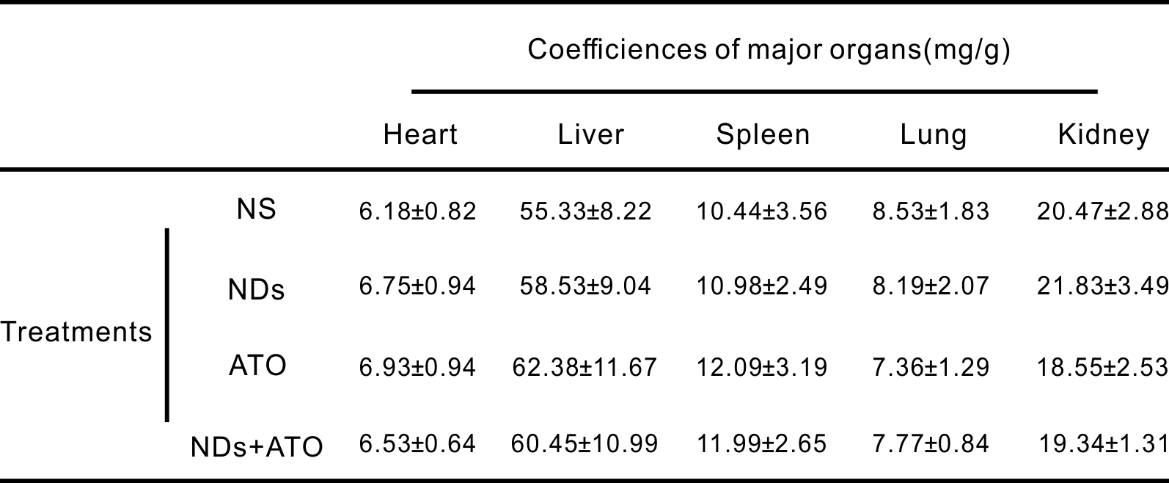


**Figure S1.** Coefficients of vital organs in subcutaneous xenograft mice after different treatments.

**
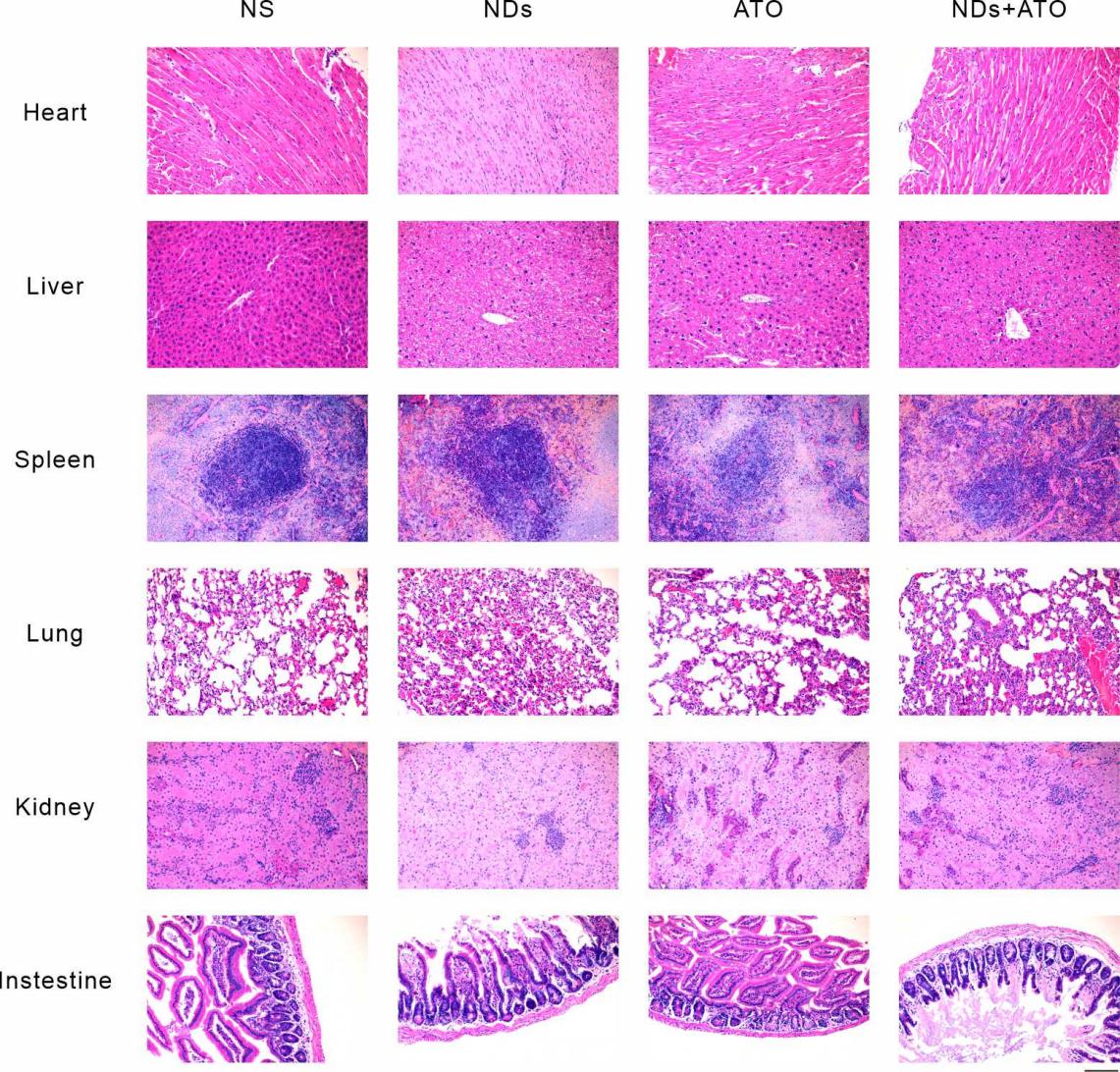
**

**Figure S2.** The HE staining of vital organs in subcutaneous xenograft mice after different treatments. Scale bar:100 μm.


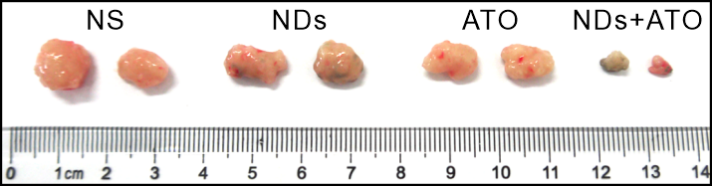


**Figure S3.** Images of excised tumors in different treatment groups.
